# Age-Based Anthropometric Cutoffs Provide Inconsistent Estimates of Undernutrition: Findings from a Cross-Sectional Assessment of Late-Adolescent and Young Women in Rural Pakistan

**DOI:** 10.1093/cdn/nzab130

**Published:** 2021-11-10

**Authors:** Jo-Anna B Baxter, Jean-Luc Kortenaar, Yaqub Wasan, Amjad Hussain, Sajid B Soofi, Imran Ahmed, Zulfiqar A Bhutta

**Affiliations:** Centre for Global Child Health, Hospital for Sick Children, Toronto, Ontario, Canada; Department of Nutritional Sciences, University of Toronto, Toronto, Ontario, Canada; Centre for Global Child Health, Hospital for Sick Children, Toronto, Ontario, Canada; Centre of Excellence in Women and Child Health, Aga Khan University, Karachi, Pakistan; Centre of Excellence in Women and Child Health, Aga Khan University, Karachi, Pakistan; Centre of Excellence in Women and Child Health, Aga Khan University, Karachi, Pakistan; Centre of Excellence in Women and Child Health, Aga Khan University, Karachi, Pakistan; Centre for Global Child Health, Hospital for Sick Children, Toronto, Ontario, Canada; Department of Nutritional Sciences, University of Toronto, Toronto, Ontario, Canada; Centre of Excellence in Women and Child Health, Aga Khan University, Karachi, Pakistan; Dalla Lana School of Public Health, University of Toronto, Toronto, Ontario, Canada

**Keywords:** body mass index, stunting, adolescent girl, young woman, undernutrition, thinness, underweight, overweight, obesity, anthropometry

## Abstract

Ambiguity around age ranges for adolescence and adulthood can make the application of age-based nutrition cutoffs confusing. We examined how estimates generated using the age-based anthropometric cutoffs for adolescent girls (10 to <19 y) and women of reproductive age (15–49 y) compared between late-adolescent and young women, and determined how application of both cutoffs affected late-adolescents’ estimates. Using cross-sectional data from participants aged 15–23 y in the Pakistan-based Matiari emPowerment and Preconception Supplementation (MaPPS) Trial (*n* = 25,447), notably large differences in estimates were observed for stunting (30.5% and 7.9% for late-adolescent and young women, respectively; *P* < 0.001) and thinness (9.3% and 30.8%, respectively; *P* < 0.001). When both cutoffs were applied to adolescents’ data, estimate differences were maintained. With each year of age, the difference for stunting increased and thinness decreased. Given the discrepancies observed both between and within groups, clarity around application of anthropometric cutoffs for youth (aged 15–24 y) is needed.

This trial was registered at clinicaltrials.gov as NCT03287882.

## Teaser

Among late-adolescent and young women, the age-based anthropometric cutoffs yield estimates with a large magnitude of difference between and within groups for indicators of undernutrition, particularly thinness and stunting.

## Introduction

Malnutrition is a condition that results from consuming a diet with too few, too many, or imbalanced energy and/or nutrients, such that health problems can arise. Among adolescent girls, malnutrition can result in adverse health events and poor reproductive outcomes, with potential intergenerational consequences (1). There is a lack of consensus among international bodies around what age range the term “adolescent” corresponds to, as well as when to use terms such as “child,” “young people,” or “youth” (see **Supplemental Table 1**) (2). Adding to the complexity, the classification “women of reproductive age” (WRA; commonly considered 15–49 y) also overlaps with “adolescent” (commonly considered 10–19 y) (3). Nonetheless, these terms are crucial to national and international demographic data reporting and research, which collectively serve to inform public health priorities and policies.

Measuring the physical dimensions of the body, called anthropometric assessment, is a relatively nonintrusive and economical way to assess nutritional status, and age-based cutoffs have been established. In low- and middle-income settings, where country-specific growth standards do not exist, knowing which age-based cutoffs to apply for certain nutritional status–related indicators can be confusing if a population straddles age categories. Of particular note is the application of the relevant anthropometric indexes for thinness and stunting, which serve as acute and chronic measures of undernutrition, respectively. For adolescent girls (aged 10 to <19 y) and adult women (aged ≥20 y), use of these indexes can lead to vastly different estimations of the burden of undernutrition (4). The adolescent cutoffs are based on the application of statistical probabilities to the WHO Growth Reference curves, which were generated to fit with the adult cutoffs for overweight and obesity (5); whereas the adult cutoffs correspond to health risks associated with being underweight and overweight (see **Supplemental Table 2**) (6).

In South Asia, the prevalence of undernutrition is high among adolescent girls and WRA (7). Within our study population in Pakistan, which included adolescent girls and young women (aged 15–23 y at enrollment), we aimed to examine how *1*) prevalence estimates from the application of the age-based cutoffs for stunting, thinness, overweight, and obesity compared between adolescent and young women; and *2*) applying both of the WHO anthropometric cutoffs affected prevalence estimates among adolescent participants aged 15–18 y, given the overlap in age with WRA (15–49 y of age).

## Methods

We used anthropometric data collected cross-sectionally from late-adolescent (aged 15–18 y; *n* = 14,771) and young women (aged 19–23 y; *n* = 10,676) upon enrollment in the Matiari emPowerment and Preconception Supplementation (MaPPS) Trial in Pakistan (NCT03287882) (8). The MaPPS Trial is a population-based, 2-arm, cluster-randomized, controlled trial of life skills building education and multiple micronutrient supplementation among adolescent and young women (aged 15–23 y at enrollment) in Matiari District. As a population-based effectiveness study, the MaPPS Trial used broad eligibility criteria to improve the generalizability of results. Adolescent and young women were not included if they reported being currently pregnant, participating in a different nutrition trial, or intending to leave the trial area. The sample size was based on the primary outcome: low-birth-weight births. In order to observe enough pregnancies for a 25% relative reduction in low-birth-weight births, the MaPPS Trial aimed to recruit ∼25,400 nonpregnant adolescent and young women. Participants were recruited from June 2017 to July 2018. Ethical review for the MaPPS Trial was obtained from the Aga Khan University Ethics Review Committee (Protocol #4324-Ped-ERC-16) and the Hospital for Sick Children Research Ethics Board (Protocol #1000054682).

Anthropometric measures, including weight and height, were collected in duplicate by 2 trained data collectors using a digital floor scale (Seca 813; Seca) and stadiometer (Seca 213; Seca), respectively. If the discrepancy between the 2 measurements exceeded the preset allowable difference (weight: <0.5 kg; height: <1.0 cm), a third measurement was obtained. The mean of acceptable measures was used in all analyses. BMI was calculated from the mean acceptable weight and height measures. Because these measures were collected at enrollment (i.e., before the provision of the intervention), all data have been considered cross-sectionally.

To estimate the number of participants affected by different forms of malnutrition, standard cutoffs were applied to their anthropometric measures (see Supplemental Table 2). For adolescent participants’ height and weight measures, the WHO igrowup package for Stata (Stata Corporation) was used to generate height-for-age *z* scores (HAZs) and BMI-for-age *z* scores (BAZs), and the WHO Growth Reference cutoffs were applied to create prevalence estimates for stunting and BAZ categories (5). The WHO Expert Committee anthropometric cutoffs for height and BMI were applied to all participant data (6, 9). Although originally intended for adults (≥20 y of age), these cutoffs are standardly used for WRA (15–49 y of age) within international reporting (3).

After applying both sets of cutoffs, we further investigated adolescent participants’ under- and overnutrition estimates by completed year of age, and calculated the absolute percentage point difference between the estimates. Estimates for the anthropometric indicators have been presented as a percentage, and continuous anthropometric outcomes as mean ± SD. To test whether there was a difference in estimates between the 2 age groups (late-adolescent and young women), a chi-square test or *t* test was used for proportions and continuous measures, respectively. A chi-square test was also used to test for differences between estimates by year of completed age using data from the late-adolescent participants.

## Results

We found that the occurrence of various forms of undernutrition was high among late-adolescent and young women enrolled in the MaPPS Trial (**Table 1**). There were large differences in estimates when using the comparative age-based cutoffs, particularly for stunting (30.5% and 7.9% for late-adolescent and young women, respectively; *P* < 0.001) and thinness (9.3% and 30.8%, respectively; *P* < 0.001). Differences between the estimates for overweight and obesity were also observed.

**TABLE 1:** Prevalence of stunting and BMI-related characteristics among late-adolescent girls and young women enrolled in the Matiari emPowerment and Preconception Supplementation (MaPPS) Trial at enrollment when applying the age-based cutoffs^1^

Characteristic	15–18 y (*n* = 14,771)	19–23 y (*n* = 10,676)	Difference between groups^2^	*P* value
Weight, kg	45.6 ± 8.5	48.9 ± 10.2	3.3 kg	<0.001
Height, cm	152.1 ± 5.7	153.0 ± 5.7	0.9 cm	<0.001
Stunted^3^	4508 (30.5)	840 (7.9)	22.6%	<0.001
BMI, kg/m^2^	19.7 ± 3.3	20.9 ± 4.0	1.2 kg/m^2^	<0.001
BMI-related categorizations				
Thinness^4^	1374 (9.3)	3291 (30.8)	21.5%	<0.001
Overweight^5^	1011 (6.8)	1138 (10.7)	3.9%	<0.001
Obese^6^	250 (1.7)	381 (3.6)	1.9%	<0.001

1 Values are mean ± SD or *n* (%) unless otherwise indicated.

2 Determined from prevalence at 15–18 y compared with 19–23 y.

3 To determine stunting, HAZ < −2 SD was used for 15–18 y and height <145.0 cm for 19–23 y.

4 To determine thinness (also referred to as underweight in adult populations), BAZ < −2 SD was used for 15–18 y and BMI <18.5 for 19–23 y.

5 To determine overweight, 1 SD < BAZ < 2 SD was used for 15–18 y and BMI 25.0–29.9 for 19–23 y.

6 To determine obese, BAZ ≥2 SD was used for 15–18 y and BMI ≥30.0 for 19–23 y.

When both sets of cutoffs were applied to late-adolescent participants’ anthropometric measures, there was an absolute difference in the prevalence of stunting (20.3%, *P* < 0.001), which increased with each year of age (**Table 2**). This corresponded to a decrease in the proportion of participants with a height <145 cm by each year of age. For thinness and overweight, there was also a difference, although the proportion decreased with each year of age.

**TABLE 2:** Comparison of the application of anthropometric cutoffs to data collected at enrollment from adolescent participants in the Matiari emPowerment and Preconception Supplementation (MaPPS) Trial, including all data and breakdown by year of age^1^

	All (15–18 y) (*n* = 14,771)			15 y (*n* = 3753)			16 y (*n* = 3721)			17 y (*n* = 3407)			18 y (*n* = 3890)		
Characteristic	GR	WRA	Δ,^2^ %	GR	WRA	Δ,^2^ %	GR	WRA	Δ,^2^ %	GR	WRA	Δ,^2^ %	GR	WRA	Δ,^2^ %
HAZ or height,^3^ cm	−1.59 ± 0.85	152.1 ± 5.7	—	−1.59 ± 0.82	151.4 ± 5.6	—	−1.59 ± 0.84	152.0 ± 5.6	—	−1.57 ± 0.84	152.6 ± 5.6	—	−1.60 ± 0.89	152.6 ± 5.8	—
Stunted^4^	4508 (30.5)	1505 (10.2)	20.3**	1125 (30.0)	456 (12.2)	17.8**	1154 (31.0)	406 (10.9)	20.1**	1008 (29.6)	283 (8.3)	21.3**	1221 (31.4)	360 (9.3)	22.1**
Weight, kg	—	45.6 ± 8.5	—	—	43.9 ± 7.8	—	—	45.2 ± 8.3	—	—	46.4 ± 8.4	—	—	47.1 ± 9.1	—
BAZ or BMI^5^	−0.64 ± 1.15	19.7 ± 3.3	—	−0.71 ± 1.11	19.1 ± 3.0	—	−0.68 ± 1.15	19.5 ± 3.3	—	−0.61 ± 1.14	19.9 ± 3.3	—	−0.58 ± 1.18	20.2 ± 3.5	—
Thinness^6^	1374 (9.3)	6105 (41.3)	−32.0**	365 (9.7)	1835 (48.9)	−39.2**	348 (9.4)	1630 (43.8)	−34.4**	306 (9.0)	1263 (37.1)	−28.1**	355 (9.1)	1377 (35.4)	−26.3**
Overweight^7^	1011 (6.8)	873 (5.9)	0.9*	215 (5.7)	151 (4.0)	1.7*	237 (6.4)	195 (5.2)	1.2*	233 (6.8)	208 (6.1)	0.7	326 (8.4)	319 (8.2)	0.2
Obese^8^	250 (1.7)	196 (1.3)	0.4*	41 (1.1)	23 (0.6)	0.5*	63 (1.7)	42 (1.1)	0.6*	64 (1.9)	55 (1.6)	0.3	82 (2.1)	76 (2.0)	0.1

1 Values are mean ± SD or *n* (%) unless otherwise indicated. BAZ, BMI-for-age *z* score; GR, values generated using 2007 WHO Growth Reference for children and adolescents aged 5 to <19 y; HAZ, height-for-age *z* score; WRA, values generated using WHO Expert Committee BMI references for adults.

2 Determined from prevalence for GR minus prevalence for WRA.

3 HAZ presented for GR; height presented for WRA.

4 GR cutoff: HAZ <−2 SD; WRA cutoff: height <145.0 cm.

5 BAZ presented for GR; BMI (in kg/m^2^) presented for WRA.

6 GR cutoff: BAZ <−2 SD; WRA cutoff: BMI <18.5 (also referred to as underweight in adult populations).

7 GR cutoff: 1 SD < BAZ < 2 SD; WRA cutoff: BMI between 25.0 and 29.9.

8 GR cutoff: BAZ ≥2 SD; WRA cutoff: BMI ≥30.0.

* *P* < 0.05, **P < 0.0001.

## Discussion

Using cross-sectional anthropometric data collected from late-adolescent girls (aged 15–18 y) and young women (aged 19–23 y), we found large discrepancies in estimates of acute and chronic undernutrition between groups using the respective age-based cutoffs, as well as within the adolescent group when both sets of cutoffs were applied. Given this inconsistency, we believe there is a need for clarity around the application of anthropometric cutoffs for youth (aged 15–24 y), especially in populations with a high prevalence of undernutrition and early pregnancies.

The large differences in the prevalence estimates for undernutrition when applying the WHO anthropometric cutoffs to our data can be attributed to a lack of alignment between the age-based indicators. The underlying reasoning for this has been well described previously in the literature (4). For stunting, HAZ < −2 SD equates to height <150.1 cm, whereas the adult cutoff is <145.0 cm (around HAZ < −3 SD); for thinness, BAZ < −2 SD equates to BMI (in kg/m^2^) <16.5, compared to <18.5 for adults (around BAZ < −1 SD). Given the close proximity of the ages of participants in our study, and that the onset of menarche was similar between our study and the population used to derive the WHO Growth Reference (13.0 compared with 12.8 y), the differences in the prevalence estimates for stunting and thinness between groups (>20%) are implausible and reflect the methodological limitations of the adolescent indicators. The findings among our Pakistani study population are in agreement with the irregularities in estimates of undernutrition observed by Tumilowicz et al. (4) among adolescent and adult women in Bangladesh. Although proportionally fewer participants in our study were overweight or obese, we found these estimates aligned more closely, likely because the WHO Growth Reference was generated to match the adult overweight and obesity cutoffs (5).

When we applied the 2 sets of cutoffs to adolescent participants’ data, discrepancies in undernutrition estimates were similarly observed. With each completed year of age, mean height and weight increased, and, consequently, so did BMI. BAZ also increased, but HAZ did not change. Collectively, this suggests some ongoing physiological development (e.g., skeletal development, bone mass accrual) occurs during late adolescence in the study population (10). The relation between the adolescent cutoffs and health outcomes, particularly those important to pregnancy, is yet to be well understood. There are limited data sets with this sort of information for adolescent girls at this time; however, we aim to use prospectively collected pregnancy data from the ongoing MaPPS Trial to investigate this further.

With respect to nutrition policy and programming, the discrepancy in prevalence between cutoffs has important implications for assessing undernutrition, because use of the different standards will potentially identify different individuals. This could be considered similar to the inconsistencies between the use of weight-for-height and midupper arm circumference to diagnose acute malnutrition in children, also in that each indicator is associated with different risks (e.g., mortality) (11). Within Pakistan, specifically, the current governmental strategy for adolescent nutrition suggests using HAZ and BAZ cutoffs to identify those who are stunted and underweight, respectively (12).

Ultimately, it has been suggested that the lack of coordination between cutoffs reflects that the adolescent indicators for stunting and thinness have limited meaning because they do not reflect health outcomes (4). Notably, adult cutoffs for short stature and low BMI are associated with increased risk of adverse birth outcomes and obstetric risk (7). Adult anthropometric measures are not without their limitations, particularly BMI because it lacks sensitivity to excess weight due to adiposity as opposed to muscularity or edema (13). However, between the *1*) overlap in age range between adolescents and WRA; *2*) call for increased nutrition data on young people (14); and *3*) potential for growth in late adolescence (15), further clarity on how to apply relevant anthropometric cutoffs would strengthen the presentation and interpretation of the corresponding indicators.

## Supplementary Material

nzab130_Supplemental_File

## Data Availability

The anonymized, individual-level data from this cross-sectional assessment, which was situated within a clinical trial, will be made available upon reasonable request from the corresponding author.

## Notes

Funding for the MaPPS Trial was provided by Bill and Melinda Gates Foundation grant OPP1148892 (to ZAB) and World Food Programme grant HQ15NF493-CTR (to ZAB). The funders had no involvement in the study design, data collection, analysis and interpretation of the data, writing of the report, or decision to submit the manuscript for publication.

Author disclosures: The authors report no conflicts of interest.

Supplemental Tables 1 and 2 are available from the “Supplementary data” link in the online posting of the article and from the same link in the online table of contents at https://academic.oup.com/cdn/.

Abbreviations used: BAZ, BMI-for-age *z* score; HAZ, height-for-age *z* score; MaPPS, Matiari emPowerment and Preconception Supplementation; WRA, women of reproductive age.
