# Supplementary material for: Age-Based Anthropometric Cutoffs Provide Inconsistent Estimates of Undernutrition: Findings from a Cross-Sectional Assessment of Late-Adolescent and Young Women in Rural Pakistan
Source: Curr Dev Nutr. 2021 Nov 10;5(11):nzab130. doi: 10.1093/cdn/nzab130 (PMC8656149; doi:10.1093/cdn/nzab130)
Supplement: nzab130_Supplemental_File [file nzab130_Supplemental_File.docx]

**Age-based Anthropometric Cut-Offs Provide Inconsistent Estimates of Undernutrition: Findings from a Cross-Sectional Assessment of Late Adolescent and Young Women in Rural Pakistan**

Jo-Anna B. Baxter, Jean-Luc Kortenaar, Yaqub Wasan, Amjad Hussain, Sajid B. Soofi, Imran Ahmed, Zulfiqar A. Bhutta

**Online Supplementary Material**

Supplemental Table 1. Terms and age categorizations for adolescence and young adulthood that appear in the literature

| **Term that appears in the literature** | **Corresponding age range**  (years) | **Reference** |
| --- | --- | --- |
| Child | <18 | UNGA 1989 [1] |
| Adolescent | 10-19 | WHO 2014 [2] |
| Early adolescent | 10-14 | WHO 2014 [2] |
| Late adolescent | 15-19 | WHO 2014 [2] |
| Adolescent | 10-24 | Sawyer et al 2012 [3] |
| Young people | 10-24 | WHO 2014 [2] |
| Youth | 15-24 | UNGA 1989 [1] |
| Women of reproductive age | 15-49 | Croft et al 2018 [4] |
| Adult woman | ≥20 | WHO 1995 [6] |

Supplemental Table 2. Anthropometric cut-offs used within different international reporting standards

| **Indicator** | **WHO Growth Reference cut-off for adolescent girls**  (15-18 years) | **WHO anthropometric cut-offs for adult women**  (≥20 years) | **Demographic Health Survey anthropometric cut-offs for WRA**  (15-49 years) |
| --- | --- | --- | --- |
| Stunting | HAZ <-2 SD | Height <145 cm | Height <145 cm |
| Thinness/underweight | BAZ <-2 SD | BMI <18.5 kg/m^2^ | BMI <18.5 kg/m^2^ |
| Overweight | BAZ > 1 SD | 25.0 < BMI < 29.9 kg/m^2^ | 25.0 < BMI < 29.9 kg/m^2^ |
| Obese | BAZ > 2 SD | BMI ≥30.0 kg/m^2^ | BMI ≥30.0 kg/m^2^ |
| Reference | de Onis et al 2007 [5] | WHO 1995 [6],  Garcia and Mason 1992 [7] | Croft et al 2018 [4] |

**References**

1. United Nations General Assembly. Convention on the rights of the child, Treaty Series, 1577:3. New York: United Nations; 1989.
2. World Health Organization. Health for the World’s Adolescents: A second chance in the second decade [Internet]. Geneva: World Health Organization; 2014 [Accessed 14 March 2020]. Available from: [www.who.int/adolescent/second-decade](http://www.who.int/adolescent/second-decade)
3. Sawyer SM et al. Adolescence: a foundation for future health. The Lancet. 2012;379(9826):1630–40.
4. Croft, Trevor N., Aileen M. J. Marshall, Courtney K. Allen, et al. Guide to DHS Statistics. Rockville, Maryland, USA: ICF; 2018.
5. de Onis M, Onyango AW, Borghi E, Siyam A, Nishida C, Siekmann J. Development of a WHO growth reference for school-aged children and adolescents. Bull World Health Organ. 2007;85(9):660–667.
6. World Health Organization. Physical status: the use and interpretation of anthropometry. Geneva: World Health Organization; 1995.
7. Garcia M, Mason J. Second Report on the World Nutrition Situation – Volume I: Global and Regional Results. A Report compiled From Information Available to the United Nations Agencies of the ACC/SCN. Geneva: World Health Organization; 1992.
